# Cytosolic Copper Binding by a Bacterial Storage Protein and Interplay with Copper Efflux

**DOI:** 10.3390/ijms20174144

**Published:** 2019-08-25

**Authors:** Jaeick Lee, Christopher Dennison

**Affiliations:** Institute for Cell and Molecular Biosciences, Newcastle University, Newcastle upon Tyne NE2 4HH, UK

**Keywords:** copper, copper homeostasis, copper toxicity, copper storage, copper storage proteins (Csps), CopA, *Escherichia coli*

## Abstract

*Escherichia coli* has a well-characterized copper (Cu) transporting ATPase (CopA) that removes this potentially toxic metal ion from the cytosol. Growth of the strain lacking CopA (Δ*copA*) is inhibited above 0.5 mM Cu, whilst a similar effect does not occur in wild type (WT) *E. coli* until over 2.5 mM Cu. Limited expression of CopA can restore growth to WT levels in Δ*copA* *E. coli* in the presence of Cu. To study the influence of a bacterial cytosolic Cu storage protein (Csp3) on how *E. coli* handles Cu, the protein from *Bacillus subtilis* (*Bs*Csp3) has been overexpressed in the WT and Δ*copA* strains. *Bs*Csp3 can protect both strains from Cu toxicity, promoting growth at up to ~1.5 and ~3.5 mM Cu, respectively. Higher levels of Csp3 expression are needed to provide resistance to Cu toxicity in Δ*copA* *E. coli*. At 1.5 mM Cu, *Bs*Csp3 purified from Δ*copA* *E. coli* binds up to approximately four equivalents of Cu(I) per monomer. A similar number of Cu(I) equivalents can be bound by *Bs*Csp3 purified from WT *E. coli* also grown at 1.5 mM Cu, a concentration that does not cause toxicity in this strain. Much lower amounts of *Bs*Csp3 are produced in WT *E. coli* grown in the presence of 3.4 mM Cu, but the protein still counteracts toxicity and is almost half loaded with Cu(I). Csp3s can protect *E. coli* from Cu toxicity by sequestering cuprous ions in the cytosol. This appears to include an ability to acquire and withhold Cu(I) from the main efflux system in a heterologous host.

## 1. Introduction

A novel family of proteins (the Csps) that can bind and store large quantities of copper (Cu), mainly via Cys residues pointing into the cores of their four-helix bundles, have been discovered in the methanotroph *Methylosinus trichosporium* OB3b [1,2,3,4,5]. There are three Csps in this methane-oxidizing bacterium, with *Mt*Csp1 and *Mt*Csp2 predicted to be secreted from the cytosol via the twin-arginine translocase (Tat) system [6], i.e., folded and potentially with Cu(I) bound, whilst *Mt*Csp3 is cytosolic. The *Mt*Csp1 gene is upregulated upon increasing Cu in a similar manner to genes encoding subunits of the enzyme it stores Cu for [1,4]; the particulate (membrane-bound) methane monooxygenase [7]. Csps are found in approximately 40% of methanotrophs, and are present in a range of other bacteria, with the most widespread and abundant being those that lack a signal peptide, i.e., Csp3s [1,2,4].

The safe handling of Cu is particularly problematic in the reducing cytosol as Cu(I) can be highly toxic if not carefully regulated [8,9]. For many years this has been associated with reactive oxygen species formation, but more recently it has become clear that a major mechanism of toxicity is driven by the ability of Cu(I) to readily displace, or bind at locations for, the native cofactor in other metalloproteins, probably driven by its higher affinities for these sites [10]. The primary target of mismetallation appears to be enzymes with exposed Fe-S clusters [10,11], and Cu is also able to block the assembly of these cofactors [11,12,13]. It has been suggested that bacteria evolved to deal with the potential toxicity of Cu, and particularly Cu(I), by not using enzymes requiring this metal ion in the cytosol [14,15,16]. The prevalence of Csp3s in bacteria [2,4] indicates that cytosolic Cu-handling and use in these organisms may be more complex than originally thought.

Bacteria possess systems to help regulate cytosolic Cu concentrations. The best characterized, and most widespread, involves the Cu-exporting P-type ATPase CopA [15,17,18,19,20,21,22,23,24,25,26], which can be accompanied by the cytosolic Cu metallochaperone CopZ [15,22,24,27,28]. The *cop*Z gene is absent in *Escherichia coli* [21], but it has recently been shown that a very closely related protein (called CopA(Z)) can be generated by programmed ribosomal frameshifting of the *copA* gene using the first of its two cognate metal-binding domains [26]. The Cu-regulated transcription of CopA typically involves either CueR (previously called CopR) [14,17,29], CsoR [30,31], or CopY [22,32,33]. In *E. coli* the regulator is CueR, which also controls the expression of CueO [29]; a multi-Cu oxidase exported into the periplasm that has cuprous oxidase activity [21,34].

Studies in *Streptomyces lividans* have shown that its Csp3 gene is upregulated in 400 μM Cu [35], and the protein has been suggested to provide additional protection against Cu toxicity [36]. In *Streptomyces coelicolor* the Csp3 gene is downregulated in germinating spores that were obtained in 80 μM Cu [37]. The *M. trichosporium* OB3b [7] and *Pseudomonas aeruginosa* [38] Csp3s do not respond to elevated Cu levels. It therefore seems unlikely that cytosolic Csp3s perform a role solely in protecting against Cu toxicity. Rather, the safe storage of Cu(I) for target enzymes, as demonstrated for the exported *Mt*Csp1 [1], potentially also including delivery of the metal ion, would appear to be their most likely function, implying a previously unidentified requirement for Cu in the cytosol. To store Cu(I) in this compartment Csp3s need to be able to compete with, and maintain their store of Cu(I) in the presence of, other homeostasis proteins, including efflux systems. Metalloproteins that are secreted via the Tat system are largely thought to bind their cofactor prior to export [6,16], and therefore acquiring Cu(I) in the presence of cytosolic detoxification systems could also be important for Csp1s. However, CueO is Tat exported, but only in a partially folded state, and is unable to acquire Cu in the cytosol of *E. coli* in the absence of its signal peptide, even when overexpressed under conditions of Cu stress [39].

Previous work in our lab has shown that overexpression of the Csp3 from *Bacillus subtilis* (*Bs*Csp3) in the *E. coli* strain lacking CopA (Δ*copA*), and therefore also CopA(Z), facilitates growth in Cu [2]. Herein, we further investigate how Csp3s prevent Cu toxicity in *E. coli*, including analyzing their ability to bind Cu(I) in the cytosol, which is relevant for the functions these proteins perform. The chosen heterologous host (*E. coli*) is ideal for these experiments as it has a well-characterized Cu homeostasis system that does not include a Csp, and its efflux of this metal ion by CopA has been extensively studied [15,17,18,19,20,21,25,26]. Using both wild type (WT) and Δ*copA E. coli*, we have further investigated the cytosolic Cu(I)-binding ability of *Bs*Csp3, which is a tetramer of four-helix bundles that can accommodate up to ~80 Cu(I) ions [2]. This has involved analyzing the influence on growth at increasing Cu concentrations when overexpressing *Bs*Csp3 (and also *Mt*Csp3) in the cytosol. Furthermore, *Bs*Csp3 has been purified from Δ*copA* and WT *E. coli* grown at carefully selected Cu concentrations to quantify the amount of Cu(I) bound. The results obtained provide an important functional insight into Csp3s and Cu availability in the cytosol of *E. coli* under different Cu stress conditions.

## 2. Results

### 2.1. Growth of WT and ΔcopA E. coli as a Function of Cu Concentration

**Figure 1:**
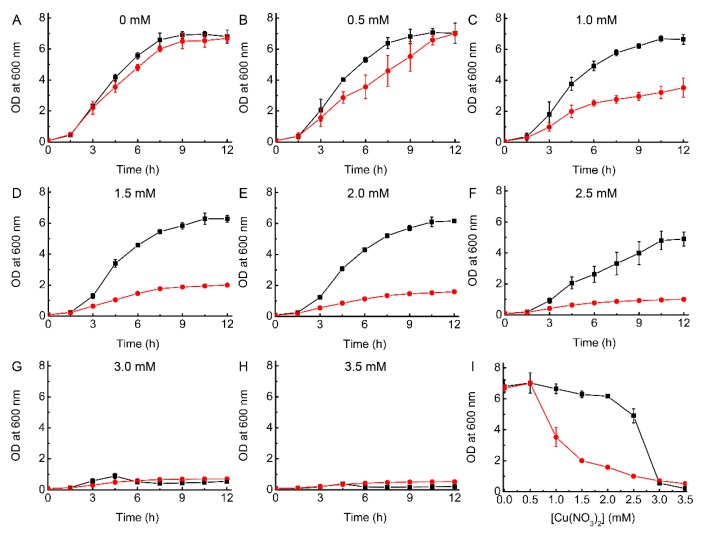
Comparison of Cu resistance in WT and Δ*copA E. coli*. Growth (37 °C) of WT (black squares) and Δ*copA* (red circles) *E. coli* in LB media plus 0 (**A**), 0.5 (**B**), 1.0 (**C**), 1.5 (**D**), 2.0 (**E**), 2.5 (**F**), 3.0 (**G**), and 3.5 (**H**) mM Cu(NO_3_)_2_. Also shown (**I**) is a comparison of growth after 12 h. The OD values are averages from two (**A**), (**B**) and (**G**), and three (**C**) to (**F**), independent growth experiments (standard deviations are shown), whilst the experiment shown in (**H**) was performed once.

The addition of Cu has a limited effect on the growth of WT *E. coli* in LB media up to a concentration of 2.5 mM (Figure 1), with the OD at 12 h changing from ~6.8 without Cu to ~4.9. However, by 3.0 mM Cu there is a dramatic decrease in growth, and the OD after 12 h only reaches ~0.5 (Figure 1G). Previous studies, also in LB media, have found similar significant effects on WT *E. coli* growth at this Cu concentration [19,20], whilst in other work ~3 mM Cu had little influence and higher concentrations were needed to cause toxicity [18,40,41]. These differences are probably related to the actual Cu concentration in the media, and because of the large effect on growth over a narrow Cu concentration range. We have therefore ensured that stock Cu(II) solutions were carefully quantified prior to use (see Materials and Methods).

Removal of the Cu pump in Δ*copA E. coli* increases Cu sensitivity (Figure 1), with 1.0 mM resulting in an OD at 12 h of ~3.5 compared to ~6.7 in the absence of added Cu (i.e., ~50% inhibition of growth). This effect is even greater when 1.5 to 2.5 mM Cu is present in the media, with the cells experiencing much more severe toxicity (OD values after 12 h of ~1.0 to ~2.0). However, growth is not completely inhibited until 3.0 mM Cu, as is also the case for WT *E. coli*. The data for Δ*copA E. coli* is also relatively consistent with previous reports [18,19,40,41], although the detailed influence of Cu concentration on growth does vary (again probably due to differences in the actual Cu concentrations used). For example, in one case, significant growth is reported at 4 mM Cu [40], whilst in another, cells failed to grow at 2.0 mM [41]. Our data indicate that very high Cu concentrations can be tolerated to some extent by *E. coli*, even in the absence of CopA, potentially due to other available mechanisms to remove the metal from the cell such as the Cus system [21].

### 2.2. Complementation Studies of ΔcopA E. coli

**Figure 2:**
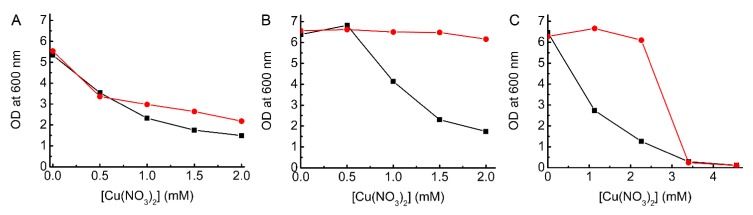
Complementation studies of Δ*copA E. coli*. Growth after 12 h (37 °C) of Δ*copA E. coli* plus pBAD33_*copA* (red circles) and pBAD33 (black squares) in LB media in the presence (**A**) and absence (**B**,**C**) of 0.2% l-arabinose at increasing concentrations of added Cu(NO_3_)_2_ (all single replicates and the data shown in (**C**) were acquired over a larger Cu(NO_3_)_2_ concentration range). Growth curves are shown in Figures S1–S3.

The overexpression of CopA in Δ*copA E. coli* induced by the addition of 0.2% l-arabinose results in similar growth at increasing Cu concentrations as for Δ*copA* transformed with pBAD33 (plasmid) alone (Figure 2A and Figure S1), and also for this strain with no plasmid (Figure 1). However, if l-arabinose is not added complementation is achieved (Figure 2B,C, Figures S2 and S3), and Δ*copA E. coli* transformed with pBAD33_*copA* grows in a similar manner to the WT strain beyond 2 mM Cu. Overall, this is consistent with previously reported data [18,40], when the caveat about variations in Cu concentrations between studies is considered. That complementation of Δ*copA E. coli* does not require induction was noted previously [18], and suggested to be due to sufficient protein being produced under these conditions to remove Cu (in the absence of an inducer some expression of CopA is expected [42]). However, the inability to complement the Δ*copA* strain in the presence of l-arabinose has not been reported. It appears that overexpression (with inducer) fails to produce a sufficient amount of an active form of this large and complex membrane protein [23].

### 2.3. Testing the Ability of BsCsp3 to Confer Resistance to Cu Toxicity in E. coli

**Figure 3:**
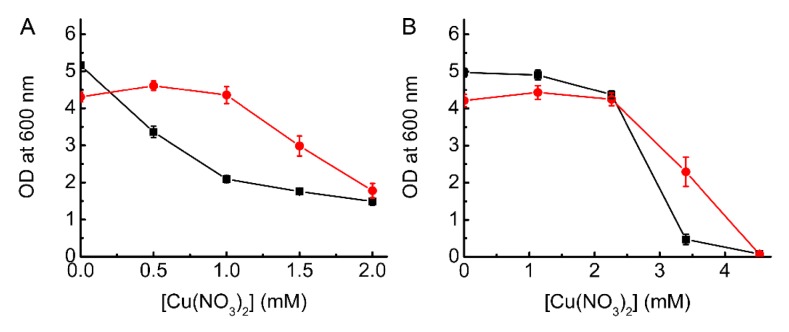
The influence of *Bs*Csp3 overexpression on the growth of Δ*copA* and WT *E. coli* in Cu. Growth (37 °C) after 12 h for Δ*copA* (**A**) and WT (**B**) *E. coli* plus pBAD33_*Bscsp3* (red circles) and pBAD33 (black squares) in LB media plus 0.2% l-arabinose in the presence of increasing concentrations of added Cu(NO_3_)_2_. Average OD values and standard deviations from three independent experiments are shown, and the growth curves are available in Figures S4 and S6, respectively.

Cytosolic overexpression of *Bs*Csp3 in *E. coli* using the same plasmid as for CopA enhances the growth of both the Δ*copA* and WT strains in the presence of toxic levels of Cu. *Bs*Csp3 improves the growth of Δ*copA E. coli* at 0.5 to 1.5 mM Cu (Figure 3A and Figure S4, these growth curves have been shown previously [2], but have not been discussed in any detail), but not as effectively as in the complemented strain, particularly beyond 1 mM Cu (Figure 2B,C). Relatively high expression of *Bs*Csp3 at 0.5 and 1.0 mM Cu (Figure S5 and Table S1) enables growth to continue beyond 4.5 h (Figure S4), which does not happen in plasmid-only controls, reaching ODs at 12 h of ~4.6 and ~4.4, respectively (Figure 3A). These are very similar to the OD obtained in the absence of added Cu (~4.3), whilst the value for the plasmid-only control remains at ~2.1 from 4.5–12 h at 1 mM Cu (i.e., *Bs*Csp3 gives a ~2.1-fold improvement in growth at this Cu concentration). At 1.5 mM Cu, the overexpression of *Bs*Csp3 is almost two-fold lower than at 1.0 mM in Δ*copA E. coli* (Figure S5 and Table S1), yet the protein can still minimize toxicity with the OD at 12 h increasing from ~1.8 in plasmid-only controls to ~3.0 (1.7-fold enhanced growth).

The overexpression of *Bs*Csp3 also provides a benefit to growth in WT *E. coli* when Cu becomes toxic, which occurs above 2.8 mM (Figure S6). The most significant advantage was found at 3.4 mM Cu (Figure 3B) with an OD after 12 h of ~2.3 compared to ~0.5 in plasmid-only controls. This is despite the low expression levels of *Bs*Csp3 in the WT strain at this Cu concentration (Figure S7 and Table S2).

**Figure 4:**
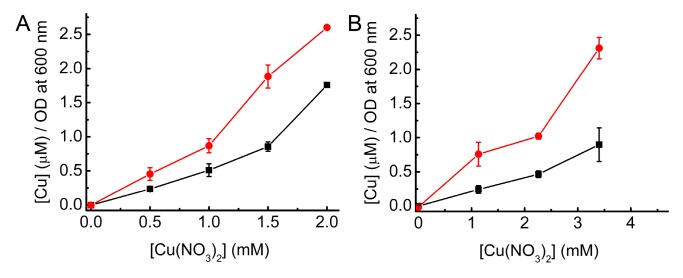
The influence of overexpressing *Bs*Csp3 on Cu levels in *E. coli*. (**A**) The Cu concentration in Δ*copA E. coli* plus pBAD33_*Bscsp3* (red circles) compared to plasmid-only controls (black squares) grown for 12 h in 0, 0.5, 1.0, 1.5 and 2.0 mM Cu(NO_3_)_2_. (**B**) Results for WT *E. coli* plus pBAD33_*Bscsp3* (red circles) and plasmid-only controls (black squares) grown for 12 h in 0, 1.1, 2.3, and 3.4 mM Cu(NO_3_)_2_ (Cu was not quantified in cells at 4.5 mM Cu(NO_3_)_2_ due to the lack of significant growth). Average values and standard deviations from three independent experiments are shown, apart from at 2.0 mM in (**A**) where the amount of Cu was measured once.

Cells producing *Bs*Csp3 acquire more Cu than plasmid-only controls (Figure 4), and the influence of overexpression on preventing Cu-induced toxicity generally corresponds with increased intracellular Cu concentration. The largest difference in Cu accumulation (~2.2-fold) for Δ*copA E. coli* is found at 1.5 mM added Cu (Figure 4A). In WT *E. coli* Cu levels are approximately two- to three-fold greater than plasmid-only controls (Figure 4B) in cells overexpressing *Bs*Csp3 at non-toxic Cu concentrations (1.1 to 2.3 mM). At 3.4 mM Cu, which has a dramatic effect on cell growth (Figure 1 and Figure 3B), a similar increase in acquired Cu (~3-fold) is observed when expressing the protein (Figure 4B). For plasmid-only controls, the Cu concentrations are greater for Δ*copA E. coli* than in the WT strain for cells grown in the presence of comparable amounts of Cu (Figure 4), consistent with limited data that is available in the literature (for example, see reference [43]).

### 2.4. Testing the Ability of MtCsp3 to Confer Resistance to Cu Toxicity in E. coli

**Figure 5:**
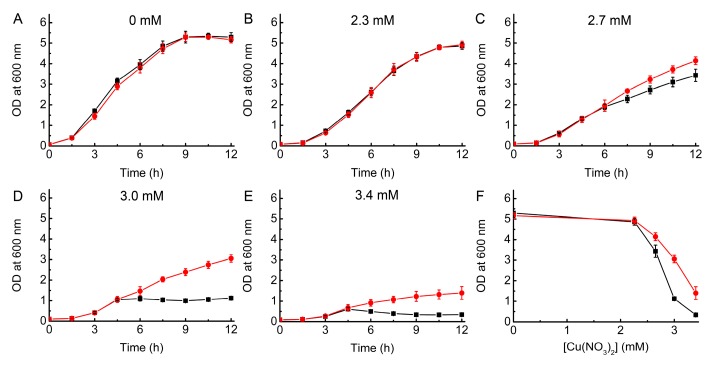
The influence of *Mt*Csp3 overexpression on the growth of WT *E. coli* in Cu. Growth (37 °C) of WT *E. coli* plus pBAD33_*Mtcsp3* (red circles) and pBAD33 (black squares) in LB media plus 0.2% l-arabinose in the presence of 0 (**A**), 2.3 (**B**), 2.7 (**C**), 3.0 (**D**), and 3.4 (**E**) mM Cu(NO_3_)_2_. A comparison of growth after 12 h is given in (**F**). Average OD values and standard deviations from three independent experiments are shown.

The overexpression of *Mt*Csp3 (the Csp3 from *M. trichosporium* OB3b) does not benefit the growth of Δ*copA E. coli* upon increasing Cu concentration (Figure S8). The levels of *Mt*Csp3 produced in Δ*copA E. coli* are lower than those of *Bs*Csp3, and decrease more significantly as Cu is added (Figures S5 and S9 and Tables S1 and S3). However, *Mt*Csp3 facilitates the growth of WT *E. coli* in a similar Cu concentration range as found for *Bs*Csp3 (Figure 5), and is expressed at a comparable level (Figures S7 and S10 and Tables S2 and S4).

### 2.5. Investigating Intracellular Cu Binding by BsCsp3 in E. coli

**Figure 6:**
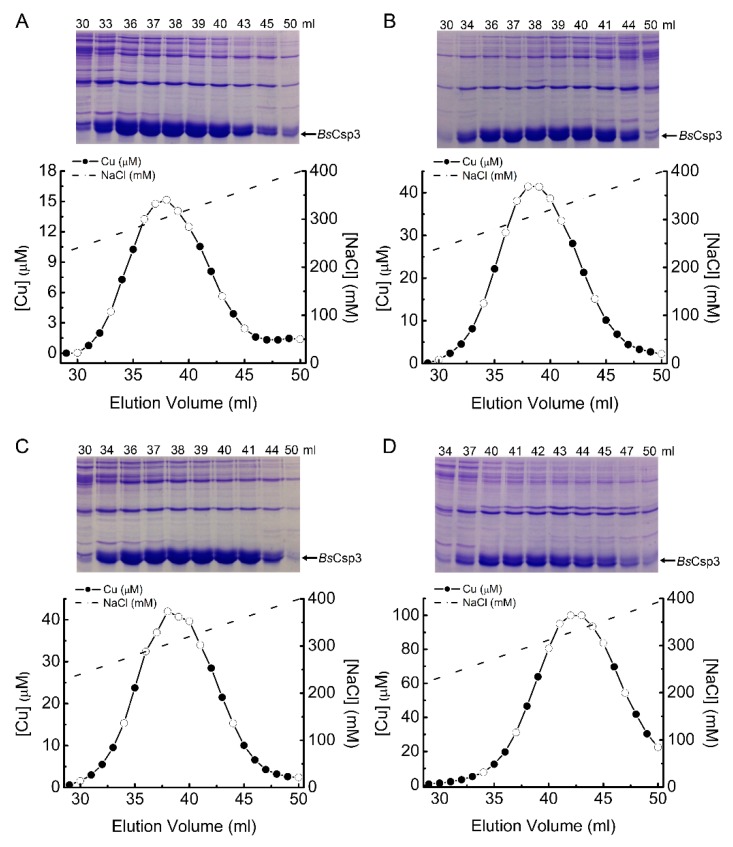
Comparison of Cu and protein contents in cell-free extracts of *E. coli* overexpressing *Bs*Csp3. The quantification of Cu in fractions that elute between approximately 230–400 mM NaCl from a HiTrap Q anion-exchange column in 20 mM Tris pH 8.0 when *Bs*Csp3 is purified from Δ*copA E. coli* grown in 1.0 (**A**) and 1.5 (**B**) mM Cu(NO_3_)_2_, and from WT *E. coli* grown in 1.5 (**C**) and 3.4 (**D**) mM Cu(NO_3_)_2_ are shown. Those fractions whose Cu concentration is indicated with open circles were analyzed by SDS-PAGE, with the elution volume above the lane.

Experiments were carried out to determine if the Cu accumulated by Δ*copA* and WT *E. coli* is bound to *Bs*Csp3. Cell-free extracts from the Δ*copA* strain overexpressing the protein at 1.0 and 1.5 mM Cu (Figure 6A,B), and WT *E. coli* grown at 1.5 and 3.4 mM Cu (Figure 6C,D), were resolved by anion-exchange chromatography, and fractions analyzed for Cu by atomic absorption spectroscopy (AAS) and protein content by SDS-PAGE. In all cases, the Cu peak that elutes at ~270 to 350 mM NaCl matches the intensity profile of the *Bs*Csp3 SDS-PAGE band, and the large amount of Cu in these fractions must be mainly bound to this protein.

**Figure 7:**
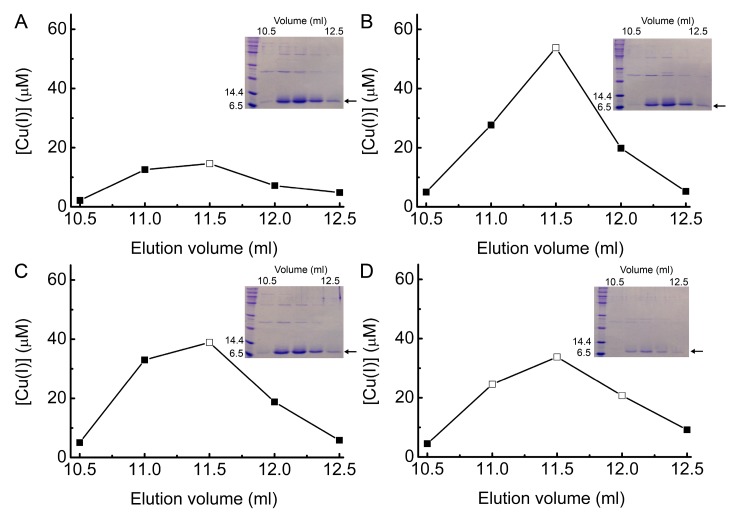
Gel-filtration chromatography of *Bs*Csp3-containing anion-exchange fractions. Plots of Cu(I) concentration against elution volume when the anion-exchange fractions that eluted at 37 and 39 mL respectively, from cell-free extracts of Δ*copA E. coli* overexpressing *Bs*Csp3 grown in 1.0 (**A**) and 1.5 (**B**) mM Cu(NO_3_)_2_ were analyzed by gel-filtration chromatography. Also shown are the gel-filtration analyses of the anion-exchange fractions eluting at 39 and 43 mL when overexpressing *Bs*Csp3 in WT *E. coli* grown in 1.5 (**C**) and 3.4 (**D**) mM Cu(NO_3_)_2_, respectively. Insets show SDS-PAGE gels confirming the main protein component in these fractions is *Bs*Csp3 (indicated by an arrow), and open squares identify which fractions were analyzed.

Gel-filtration chromatography (Figure 7 and Figure S11) was used to further purify *Bs*Csp3 present in anion-exchange fractions possessing the highest Cu concentrations from both Δ*copA* and WT *E. coli*. In all cases, the variation in Cu(I) content of the fractions for the main peak that eluted from the gel-filtration column matches the intensity profile of the *Bs*Csp3 band in SDS-PAGE analyses of these fractions (Figure 7 and Figure S11). Furthermore, this peak elutes at a very similar position to the purified *Bs*Csp3 tetramer [2], particularly when the absorbance at 240 nm against elution volume plots are considered (Figure S12). Fractions possessing large amounts of Cu were concentrated, and their purity checked by SDS-PAGE (Figure S13). The protein and Cu(I) (Figure S14) concentrations of these samples were determined and are shown in Table 1 and Figure S5. Being more selective about which fractions are combined and analyzed has a significant influence on the measured Cu(I) occupancy for *Bs*Csp3, but does not alter the relative amounts of Cu(I)-bound protein from the two *E. coli* strains grown at the two different Cu concentrations.

## 3. Discussion

There are two major outcomes form this study. Firstly, the overexpression of *Bs*Csp3 allows *E. coli* to grow at Cu concentrations not normally possible. This is true for both WT *E. coli* (*Mt*Csp3 also aids the growth of WT at similar Cu concentrations) and for the strain lacking the main Cu-efflux pump (Δ*copA E. coli*). Secondly, in cells expressing *Bs*Csp3, this protein can bind a significant amount of Cu(I). Taken together, these two observations suggest that the intracellular Csp3s can rescue cells from toxic concentrations of Cu(I) by sequestering excess cuprous ions and thereby preventing them from taking part in harmful interactions with cellular machinery. *Bs*Csp3 can also acquire Cu(I) at concentrations that are not toxic in WT *E. coli*.

The large amount of *Bs*Csp3 produced in Δ*copA E. coli* at 1 mM Cu (Figure S5 and Table S1) can overcome the toxicity caused in its absence (Figure 3A and Figure S4), yet the purified protein binds only approximately one equivalent of Cu(I) (Table 1). *Mt*Csp3 expression is lower in Δ*copA E. coli* (Figure S9 and Table S3) and does not provide any protection against Cu toxicity (Figure S8). This is consistent with the sites that can cause toxicity in Δ*copA E. coli* at 1 mM Cu having Cu(I) affinities tighter than those of Csp3s (average values of (1−2) × 10^17^ M^−1^ have been measured for Csp3s [2]), with the ability of *Bs*Csp3 to acquire Cu(I) being driven by the large amount of protein present. The locations of all sites that can result in bacterial Cu toxicity remain to be determined, although Fe-S cluster-containing proteins are currently the main target identified [8,9,10,11,12,13], and these would be expected to be able to bind Cu(I) tightly. Some Cu(I) may reside in cytosolic pools bound by a highly-abundant species such as glutathione (GSH) that has been implicated in Cu(I) handling in *E. coli* [41], although a role in toxicity is currently unclear [8,9]. The abundance of GSH in *E. coli* [44,45], along with its affinity for Cu(I) [46], is consistent with a Csp3 being able to compete with it for cuprous ions.

At 1.5 mM Cu, *Bs*Csp3 is still able to counteract toxicity in Δ*copA E. coli* (Figure 3A), even though expression levels are almost two-fold lower than at 1 mM (Figure S5 and Table S1). There is a sizable increase in the amount of Cu/cell at 1.5 mM in this strain, both in the absence and presence of *Bs*Csp3 (Figure 4A), and the purified protein can now bind up to approximately four equivalents of Cu(I) (Table 1). This increase in intracellular Cu(I) has resulted in a larger number of sites that cause toxicity, and possibly also increased levels of Cu(I) in cytosolic pools, which are more accessible to *Bs*Csp3 (higher occupancy despite lower expression), and some of the additional sites must therefore have lower Cu(I) affinities. *Bs*Csp3 appears to be able to remove Cu(I) from different cytosolic sites in Δ*copA E. coli* promoting growth. At 2 mM the intracellular Cu concentration increases further in Δ*copA E. coli* (Figure 4A), and the expression of *Bs*Csp3 is significantly lower (Figure S5 and Table S1). As a result, the protein is no longer able to provide a growth advantage compared to plasmid-only controls (Figure 3A).

The overexpression of *Bs*Csp3 in WT gives rise to lower Cu accumulation than in Δ*copA E. coli* (Figure 4 and Table S6), but the levels are higher (~2–3-fold) compared to plasmid-only controls (Figure 4B). However, unlike the drastic effect on growth observed in Δ*copA E. coli*, WT cells show no sign of experiencing toxicity in the 1.1 to 2.3 mM Cu concentration range (Figure 1 and Figure 3B). At 1.5 mM Cu, *Bs*Csp3 expression levels in WT are double those in Δ*copA E. coli* (Figures S5 and S7 and Tables S1 and S2), and the protein still acquires up to approximately four equivalents of Cu(I) (Table 1). Under these conditions, cytosolic Cu(I) is presumably being adequately handled by the efflux system (no obvious signs of toxicity). Thus, *Bs*Csp3 must be acquiring Cu(I) from different locations in WT than in Δ*copA E. coli*, with CopA being the most likely source.

In WT *E. coli*, overexpressing either *Bs*Csp3 (Figure 3B) or *Mt*Csp3 (Figure 5F) provides a benefit to growth in the relatively narrow Cu concentration window that causes toxicity, with the greatest advantage observed above approximately 2.5 mM. At these Cu concentrations, the WT strain (Figure 1), and plasmid-only control cells (Figure 3B and Figure 5), start to exhibit signs of Cu stress probably due to the efflux system becoming saturated. Although the expression levels of *Bs*Csp3 and *Mt*Csp3 are low at 3.4 mM Cu (Figures S7 and S10 and Tables S2 and S4), both proteins provide protection against Cu toxicity, enabling cells to reach an OD after 12 h that is approximately four- to five-fold higher than plasmid-only controls (Figure 3B and Figure 5).

*Bs*Csp3 purified from WT *E. coli* grown at 3.4 mM Cu can be ~50% occupied (it proved difficult to purify *Mt*Csp3, so the number of Cu(I) equivalents bound could not be determined). Saturation of the efflux system at this Cu concentration results in Cu(I) binding at sites causing toxicity. Despite their relatively low expression levels, Csp3s can acquire Cu(I) from these sites helping to prevent their harmful effects. The quantity of Cu(I) acquired by low amounts of *Bs*Csp3 (Table 1 and Table S5) indicates that in WT *E. coli* under toxicity-causing conditions the protein obtains Cu(I) from an additional source, or sources, than at 1.5 mM Cu (no toxicity), and also to those in Δ*copA E. coli*. This behavior would appear to be consistent with the proposed ability of Csp3s to act as a secondary system for buffering cellular Cu [36]. However, such functionality would be expected to require significant Cu-dependent upregulation, which is not observed in the majority of bacteria tested to date [7,35,37,38].

Another Cu(I)-binding four-helix bundle has been found to provide protection against Cu toxicity in *E. coli* [47]. This is not a naturally occurring protein, but was generated via a *de novo* design approach using a library of DNA sequences coding for proteins with this fold. The library was transformed into *E. coli*, with selection based on the ability to grow in an otherwise toxic Cu concentration. A protein, ConK, was found that can bind up to seven Cu(I) ions primarily via His residues, albeit with a relatively low (μM) affinity. The overexpression of ConK allowed WT *E. coli* (BW25113) to grow at up to 2.2 mM Cu (OD values were only measured after 20 h, and there are again uncertainties about the actual Cu concentrations used). A variant of ConK in which three of the His residues were mutated, two to Tyr and the other to Leu, enabled growth at 7 mM Cu. Overexpression of the WT protein also promoted growth at similar Cu concentrations in a strain lacking CopA and CueO, as well as the Cus [21] detoxification system. Unexpectedly, intracellular Cu levels were lowered in WT and mutant cells expressing ConK, and a mechanism that combats Cu toxicity by assisting in removal from the cytosol has been proposed [47]. How this is achieved is unknown, but could involve the transfer of Cu(I) to CopA in WT *E. coli*, which would be favored by the low affinity of ConK for Cu(I). The much tighter Cu(I) affinities of Csp3s will enable them to acquire Cu(I) from this efflux system.

## 4. Materials and Methods

### 4.1. Analysis of WT and the copA Deletion Strains of E. coli BW25113 and the Influence of BsCsp3 Overexpression

WT *E. coli* BW25113 (Coli Genetic Stock Centre, Yale University, New Haven, CT, USA, CGSC number 7636) and the strain with the *copA* gene inactivated through allelic replacement (herein called Δ*copA*; CGSG number 8625) were obtained from the CGSC library [48]. The difference between these strains was verified by PCR with the following primers; 5′-CCGATTTTTTAATCTTTACGGAC-3′ and 5′-GCGTCTTATCAGGCCTACAAACCTG-3′ designed to hybridize 100 bp upstream and downstream of the *copA* gene, giving PCR fragments of 2754 and 1571 bp, respectively (Figure S15). The *copA* gene was amplified from genomic DNA using the primers 5′-GTCCCCGCC**CATATG**TCACAAACTATCGACC-3′ (forward, *Nde*I restriction site in bold) and 5′-GGTCGTGCC**CCATGG**TTATTCCTTCGGTTTAAACCG-3′ (reverse, *Nco*I restriction site in bold, stop codon underlined). The obtained fragment was digested and cloned into the *Nde*I and *Nco*I sites of pET29a, verified by sequencing, and sub-cloned into the *Xba*I and *Hind*III sites of pBAD33 to give pBAD33_*copA*. The *Bscsp3* gene was sub-cloned from pET29a [2] into the *Xba*I and *Hind*III sites of pBAD33 to give pBAD33_*Bscsp3*.

Cultures were grown (agitation at 250 rpm) in liquid LB medium at 37 °C, and kanamycin (50 µg/mL, Formedium, Hunstanton, UK) and chloramphenicol (30 µg/mL, Merck KGaA, Darmstadt, Germany) were added as required. Overnight cultures were diluted 100-fold into LB and LB plus Cu(NO_3_)_2_ (0.5 to 4.6 mM) without antibiotics, either in the presence or absence of 0.2% l-arabinose, and the OD value at 600 nm was measured at regular intervals. For the addition of Cu, a 500 mM stock of Cu(II) nitrate trihydrate (Merck KGaA) was prepared in 100 mL of MilliQ water (Millipore Ltd., Watford, UK). After autoclaving, the Cu concentration was quantified by AAS using standards ranging from 0.2 to 1.8 ppm Cu in 2% nitric acid [49]. When cells were needed for Cu quantification and for analyzing protein expression by SDS-PAGE, ~35 and ~5 mL respectively, of a culture (50 mL in a 250 mL Erlenmeyer flask) grown for 12 h was collected by centrifugation at 5000× *g* for 10 min (4 °C) and frozen at −30 °C. Thawed cells for Cu quantification were washed twice with 20 mL of 20 mM Tris pH 8.0 plus 10 mM EDTA and 100 mM NaCl, and then with 20 mL of 20 mM Tris pH 8.0 plus 100 mM NaCl. The cells were digested in 200 μL of 65% nitric acid (Ultrapur, Merck KGaA) for up to three days at room temperature, centrifuged at 12,000× *g* for 10 min, diluted in MilliQ water to give a final nitric acid concentration of 2%, and analyzed for Cu by AAS. For SDS-PAGE analysis, thawed cells were resuspended in 3 mL of 20 mM Tris pH 8.5 and sonicated. Typically, 24 μL of this sample was taken to test for total protein expression, and the same volume was removed from the supernatant after centrifugation at 12,000× *g* for 10 min. The concentrations of *Bs*Csp3 and *Mt*Csp3 in images of SDS-PAGE gels were quantified with ImageJ using pure protein standards (18.6 μM for *Bs*Csp3 and 15.0 μM for *Mt*Csp3) that were run on the same gel.

### 4.2. Purification and Cu(I)-Binding Stoichiometry of BsCsp3

For the purification of *Bs*Csp3, 500 mL cultures were grown in 2 L Erlenmeyer flasks and harvested after growth for 12 h, during which the OD was monitored (Figure S16 and Table S6), and the resulting pellets were stored at −30 °C. Thawed cells were washed twice with 20 mL of 20 mM Tris pH 8.0 plus 10 mM EDTA and 100 mM NaCl, and then with 20 mL of 20 mM Tris pH 8.0 plus 100 mM NaCl. Washed cells were resuspended in 20 mM Tris pH 8.0, sonicated, and centrifuged at 40,000× *g* for 30 min. The supernatant was diluted five-fold with 20 mM Tris pH 8.0 and loaded onto a HiTrap Q FF anion-exchange column (1 mL, GE Healthcare Life Sciences, Little Chalfont, UK) equilibrated in the same buffer. Proteins were eluted with a linear NaCl gradient (0 to 400 mM NaCl, total volume 50 mL). The *Bs*Csp3-containing fractions were analyzed for Cu by AAS, and protein content by SDS-PAGE. Fractions containing high concentrations of Cu were further purified on a Superdex 75 10/300 GL column (GE Healthcare Life Sciences), equilibrated in 20 mM Hepes pH 7.5 plus 200 mM NaCl. The Cu(I) concentration in eluted 500 μL fractions was quantified under anaerobic conditions using the high affinity chromophoric Cu(I) ligand bathcuproine disulfonate (BCS, Merck KGaA) [1,2,49,50,51]. Oxygen-free solutions were prepared in an anaerobic chamber (Belle Technology, Weymouth, UK, [O_2_] << 2 ppm), and 50 μL of each fraction was added using a gastight syringe to a 1000 μL solution of BCS (~2.5 mM) in 20 mM Hepes pH 7.5 plus 200 mM NaCl in the presence of guanidine hydrochloride (final concentration ~6.5 M) in a sealed anaerobic cuvette. The formation of [Cu(BCS)_2_]^3−^ was monitored by UV-Vis spectrophotometry at 483 nm (ε = 12,500 M^−1^cm^−1^), typically for 2 h [1,2,49,50,51]. The protein content of these fractions was analyzed by SDS-PAGE.

To determine the number of Cu(I) equivalents bound to *Bs*Csp3, 500 μL fractions from the Superdex 75 column containing high concentrations of Cu(I)-*Bs*Csp3 were analyzed. Fractions eluting around 11.5 mL were typically chosen, and for most conditions higher occupancies were obtained using a single fraction, although the inclusion of more fractions has less of an effect in the case of samples from WT *E. coli* grown at 3.4 mM Cu. Fractions were concentrated under anaerobic conditions to 150 μL, using a Vivaspin 500 centrifugal concentrator (10 kDa molecular weight cut-off, Sartorius AG., Göttingen, Germany). The protein concentration was determined with a Bradford assay corrected using a Bradford:DTNB ratio (the 5,5′-dithiobis(2-nitrobenzoic acid) (DTNB) assay for thiols has routinely been used to measure the concentration of Csps) of 1.31 for purified apo-*Bs*Csp3 [1,2,49]. The Cu(I) concentration was determined by adding 50 μL (for some samples 12 μL was used as precipitation occurred with 50 μL) of the protein (0.46–4.81 μM) to BCS as described above.

## 5. Conclusions

The data obtained indicate that sites resulting in cellular toxicity with different Cu(I) affinities can be occupied in the cytosol of *E. coli*. Csp3s are able to compete with these, but their effectiveness can require high-level expression. The binding of Cu(I) at higher-affinity toxicity-causing sites (or bound to high-abundance species) requires greater concentrations of the apo-Csp3 to drive equilibria toward Cu(I)-Csp3. This observation, albeit in a heterologous system, provides important insight into Cu(I)-trafficking pathways. For example, as well as considering their affinities [52], the cellular concentrations of proteins need to be taken into account when assessing directional Cu (and other metal-ion) transfer within cells, as discussed previously [53]. Cytosolic Csp3s can acquire Cu(I) from the efflux system, as demonstrated by the isolation of Cu(I)-*Bs*Csp3 from WT *E. coli* at a Cu concentration that has no effect on growth.

The highest Cu(I) occupancy is observed for *Bs*Csp3 purified from WT *E. coli* experiencing Cu toxicity, and despite very low expression levels, a significant benefit to growth is provided. Under these conditions, whether Cu(I) is being acquired directly from an overloaded efflux system or from alternative sites that can cause toxicity, and have lower Cu(I) affinities to those occupied in Δ*copA E. coli*, or both, is difficult to determine. The Cu(I) removal kinetics are slow for all Csp3s studied to date [2], which should help these proteins hold onto Cu(I) they acquire until it is needed. Cu(I) removal is much faster from Csp1s, which may bind cuprous ions in the cytosol prior to Tat export [1,6]. However, CueO from *E. coli* does not bind Cu(I) when overexpressed with its Tat signal peptide removed, including in LB plus 1.5 mM Cu [39]. The behavior of this Cu-enzyme is very different from that of a cytosolic Cu storage protein (Csp3), which we show can acquire considerable amounts of Cu(I) in this compartment of *E. coli* under very similar conditions. Our studies therefore also provide important information about the Cu(I)-binding capabilities of Csp3s within the cytosol of a bacterium.

## Figures and Tables

**Table 1:** The number of Cu(I) equivalents bound by *Bs*Csp3 from the two *E. coli* strains grown at different Cu concentrations ^1^.

*E. coli* Strain and Added Cu(NO_3_)_2_ Concentration	[Cu(I)] (µM)	[*Bs*Csp3] (µM)	[Cu(I)]/[*Bs*Csp3] ^2^
Δ*copA* in 1.0 mM Cu(NO_3_)_2_	50.9	45.7	1.1
Δ*copA* in 1.5 mM Cu(NO_3_)_2_	162	39.1	4.1
WT in 1.5 mM Cu(NO_3_)_2_	166	38.7	4.3
WT in 3.4 mM Cu(NO_3_)_2_	189	20.2	9.4 ^3^

^1^ The values shown are the Cu(I) and protein concentrations for *Bs*Csp3 purified by gel-filtration chromatography (Figure 7). ^2^ Lower Cu(I) occupancies, but with the same overall pattern in the two strains grown at the different Cu(NO_3_)_2_ concentrations as above, are obtained when more fractions eluting from the gel-filtration column are combined and concentrated for another anion-exchange fraction that was analyzed (see Figure S11 and Table S5). ^3^ The protein concentration is possibly overestimated due to the lower purity of this sample (see Figure S13A), and the Cu(I) occupancy of *Bs*Csp3 could therefore be higher than the value quoted.

## Supplementary Materials

Supplementary materials can be found at https://www.mdpi.com/1422-0067/20/17/4144/s1.
