# Supplementary material for: Cytosolic Copper Binding by a Bacterial Storage Protein and Interplay with Copper Efflux
Source: Int J Mol Sci. 2019 Aug 25;20(17):4144. doi: 10.3390/ijms20174144 (PMC6747150; doi:10.3390/ijms20174144)
Supplement: Supplementary file 1 [file ijms-20-04144-s001.pdf]

SUPPLEMENTARY INFORMATION

Cytosolic Copper Binding by a Bacterial Storage Protein and Interplay with Copper Efflux

Jaeick Lee and Christopher Dennison*

Institute for Cell and Molecular Biosciences, Newcastle University, Newcastle upon Tyne, NE2 4HH, UK

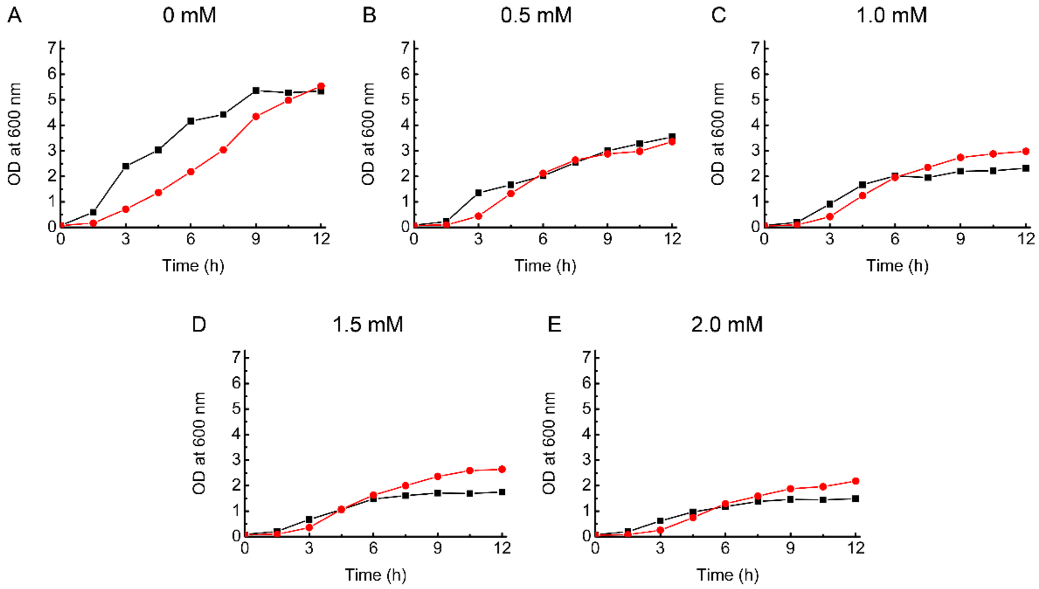

Figure S1. Complementation study of Δ*copA E. coli* in the presence of inducer. Growth (37 °C) of Δ*copA E. coli* plus pBAD33_*copA* (red circles) and pBAD33 (black squares) in LB media in the presence of 0.2% L-arabinose and 0 (A), 0.5 (B), 1.0 (C), 1.5 (D), and 2.0 (E) mM added $Cu(NO_3)_2$ corresponding to data shown in Figure 2A (single replicate).

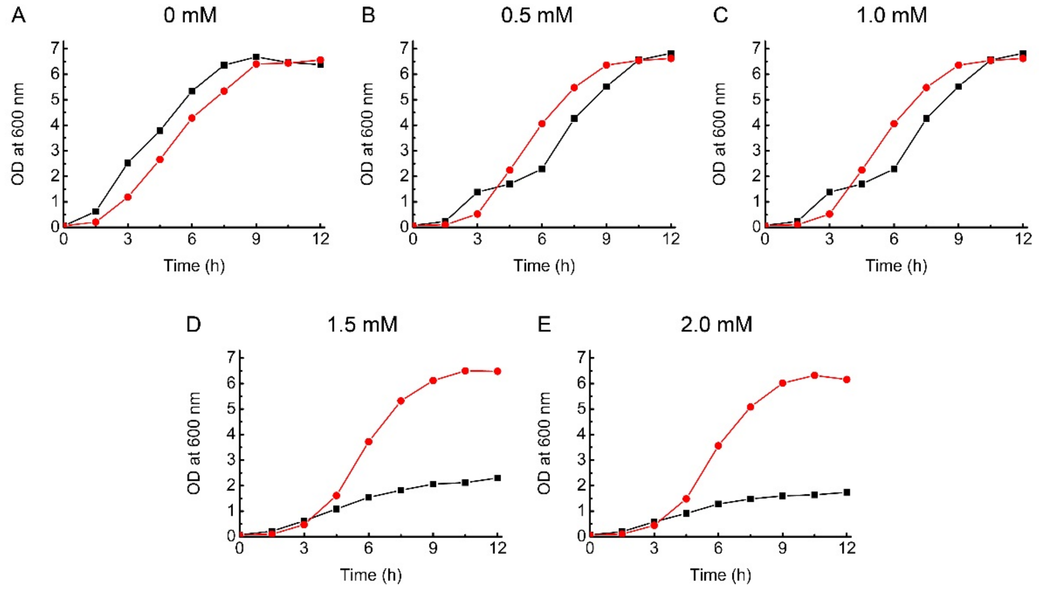

Figure S2. Complementation study of Δ*copA E. coli* in the absence of inducer. Growth (37 °C) of Δ*copA E. coli* plus pBAD33_*copA* (red circles) and pBAD33 (black squares) in LB media in the presence of 0 (A), 0.5 (B), 1.0 (C), 1.5 (D), and 2.0 (E) mM added $Cu(NO_3)_2$ corresponding to data shown in Figure 2B (single replicate).

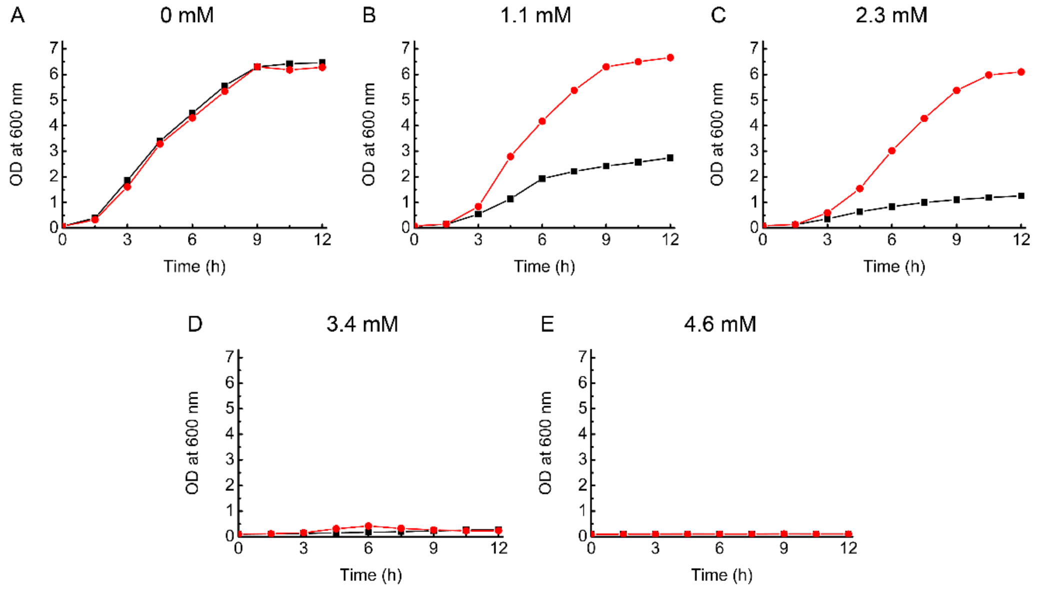

Figure S3. Complementation study of Δ*copA E. coli* in the absence of inducer. Growth (37 °C) of Δ*copA* plus pBAD33_*copA* (red circles) and pBAD33 (black squares) in LB media in the presence of 0 (A), 1.1 (B), 2.3 (C), 3.4 (D), and 4.6 (E) mM added $Cu(NO_3)_2$ corresponding to data shown in Figure 2C (single replicate).

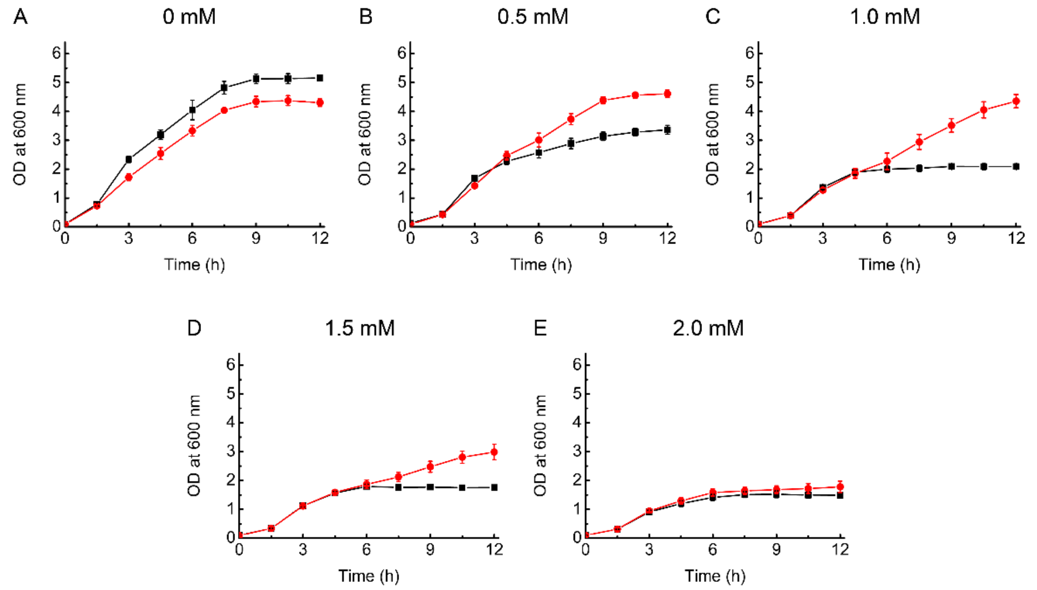

Figure S4. The influence of *Bs*Csp3 on the growth of Δ*copA E. coli* in Cu. Growth (37 °C) of Δ*copA E. coli* plus pBAD33_*Bscsp3* (red circles) and pBAD33 (black squares) in LB media plus 0.2% L-arabinose in the presence of 0 (A), 0.5 (B), 1.0 (C), 1.5 (D), and 2.0 (E) mM added $Cu(NO_3)_2$. The average OD values and standard deviations from three independent growth experiments are shown. These data have been published previously (reference [2] in the main manuscript), but have not been discussed in any detail.

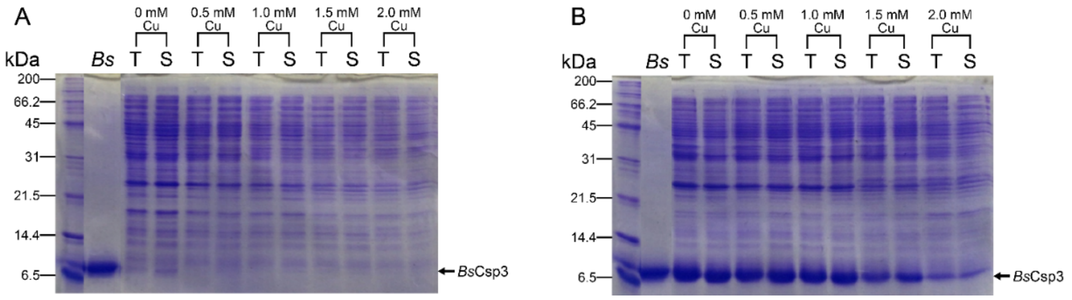

Figure S5. The influence of Cu on the expression levels of *Bs*Csp3 in Δ*copA E. coli*. Analysis by SDS-PAGE of total (T) and soluble (S) proteins in Δ*copA E. coli* plus pBAD33 (A) and pBAD33_*Bscsp3* (B) after growth for 12 h at different added $Cu(NO_3)_2$ concentrations, compared with a purified sample (18.6 μM) of *Bs*Csp3 (*Bs*).

Table S1. Quantification of *Bs*Csp3 expression levels in Δ*copA E. coli*.

$Cu(NO_3)_2$ (mM)	Proteins	[*Bs*Csp3] (μM) [1]
0	Total	34.6 ± 14.5
	Soluble	33.1 ± 10.5
0.5	Total	28.9 ± 13.1
	Soluble	31.3 ± 14.3
1.0	Total	27.4 ± 14.2
	Soluble	26.4 ± 12.1
1.5	Total	10.8 ± 6.81
	Soluble	12.4 ± 7.13
2.0	Total	2.73 ± 1.09
	Soluble	2.48 ± 1.60

[1] The concentrations of *Bs*Csp3 were calculated using the software ImageJ and the average values and standard deviations from three independent growth experiments are shown.

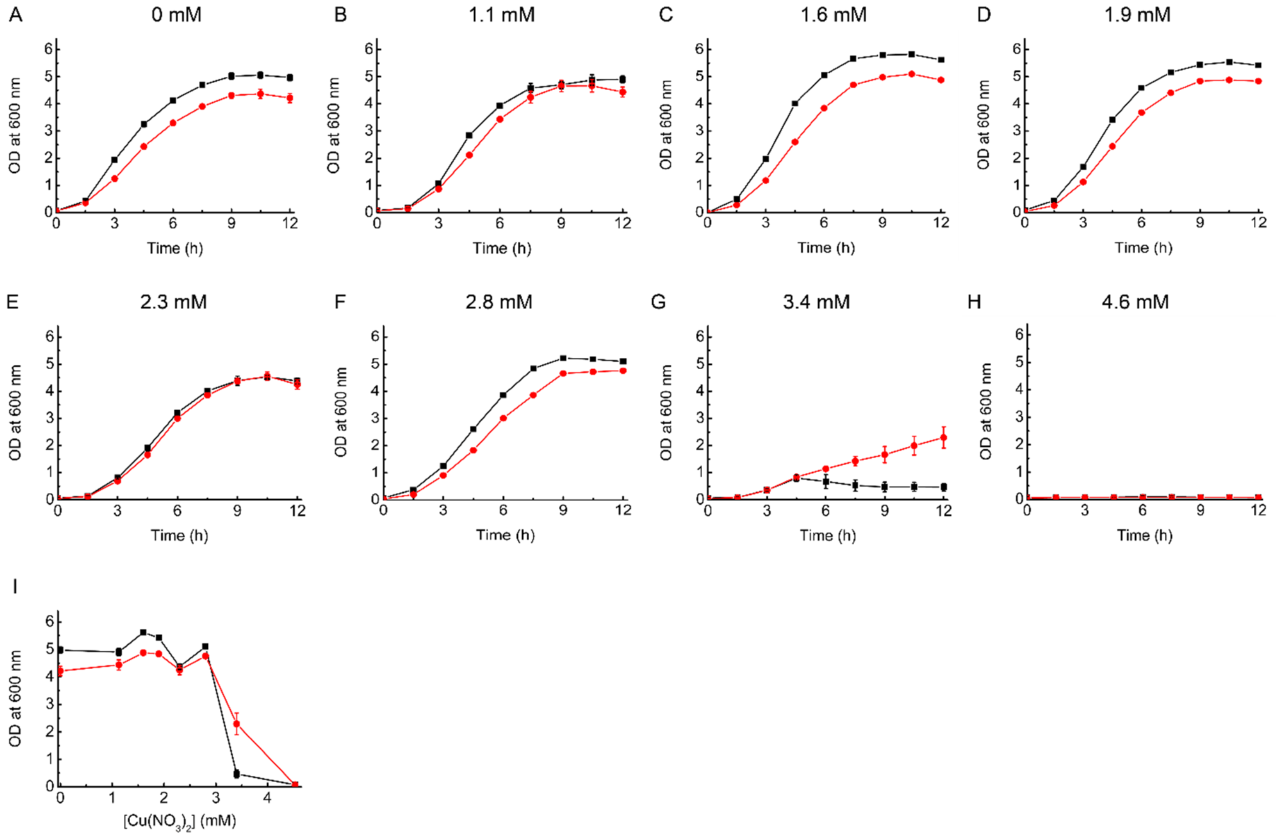

Figure S6. The influence of *Bs*Csp3 on the growth of WT *E. coli* in Cu. Growth (37 °C) of WT *E. coli* plus pBAD33_*Bscsp3* (red circles) and pBAD33 (black squares) in LB media plus 0.2% L-arabinose in the presence of 0 (A), 1.1 (B), 1.6 (C), 1.9 (D), 2.3 (E), 2.8 (F), 3.4 (G), and 4.6 (H) mM added $Cu(NO_3)_2$. The OD values are averages from three (A), (B), (E), (G) and (H), independent growth experiments (standard deviations are shown), whilst the experiments shown in (C), (D) and (F) were performed once and shown in (I) is a comparison of the OD after 12 h. The growth curves at 0 (A) and 3.4 (G) mM added $Cu(NO_3)_2$ have been shown previously (reference [4] in the main manuscript), but have not been discussed in any detail.

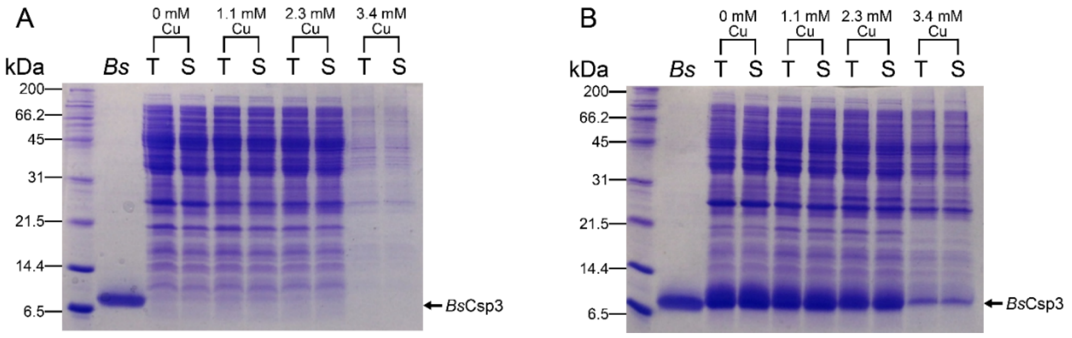

Figure S7. The influence of Cu on the expression levels of *Bs*Csp3 in WT *E. coli*. Analysis by SDS-PAGE of total (T) and soluble (S) proteins in WT *E. coli* plus pBAD33 (A) and pBAD33_*Bscsp3* (B) after growth for 12 h at different added $Cu(NO_3)_2$ concentrations, compared with a purified sample (18.6 μM) of *Bs*Csp3 (*Bs*).

Table S2. Quantification of *Bs*Csp3 expression levels in WT *E. coli*.

$Cu(NO_3)_2$ (mM)	Proteins	*Bs*Csp3 (μM) [1]
0	Total	27.6 ± 6.11
	Soluble	28.4 ± 7.57
1.1	Total	27.5 ± 9.27
	Soluble	28.1 ± 7.47
2.3	Total	23.0 ± 5.76
	Soluble	22.7 ± 5.34
3.4	Total	3.68 ± 1.01
	Soluble	3.24 ± 0.46

[1] The concentrations of *Bs*Csp3 were calculated using the software ImageJ and the average values and standard deviations from three independent growth experiments are shown.

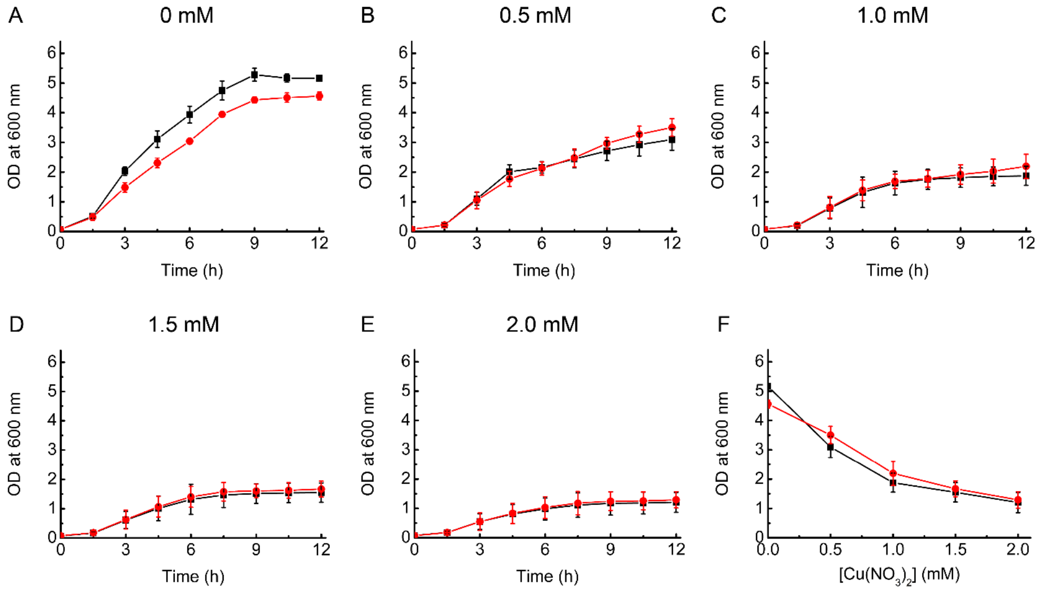

Figure S8. The influence of *Mt*Csp3 on the growth of Δ*copA E. coli* in Cu. Growth (37 °C) of Δ*copA E. coli* plus pBAD33_*Mtcsp3* (red circles) and pBAD33 (black squares) in LB media plus 0.2% L-arabinose in the presence of 0 (A), 0.5 (B), 1.0 (C), 1.5 (D), and 2.0 (E) mM added $Cu(NO_3)_2$. Also shown (F) is a comparison of the OD after 12 h. The average OD values and standard deviations from three independent growth experiments are shown.

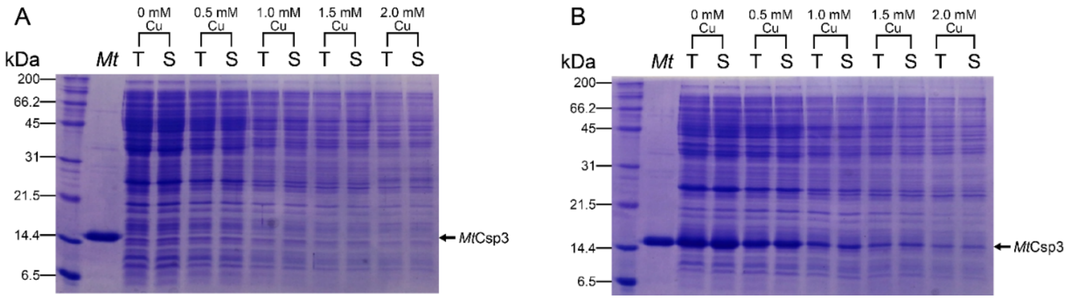

Figure S9. The influence of Cu on the expression levels of *Mt*Csp3 in Δ*copA E. coli*. Analysis by SDS-PAGE of total (T) and soluble (S) proteins in Δ*copA E.coli* plus pBAD33 (A) and pBAD33_*Mtcsp3* (B) after growth for 12 h at different added $Cu(NO_3)_2$ concentrations, compared with a purified sample (15.0 μM) of *Mt*Csp3 (*Mt*).

Table S3. Quantification of *Mt*Csp3 expression levels in Δ*copA E. coli*.

$Cu(NO_3)_2$ (mM)	Proteins	*Mt*Csp3 (μM) [1]
0	Total	15.0 ± 4.58
	Soluble	14.8 ± 5.28
0.5	Total	9.67 ± 3.34
	Soluble	9.36 ± 3.68
1.0	Total	5.17 ± 1.85
	Soluble	4.87 ± 2.18
1.5	Total	4.07 ± 1.32
	Soluble	4.12 ± 1.18
2.0	Total	2.84 ± 0.86
	Soluble	2.40 ± 0.88

[1] The concentrations of *Mt*Csp3 were calculated using the software ImageJ and the average values and standard deviations from three independent growth experiments are shown.

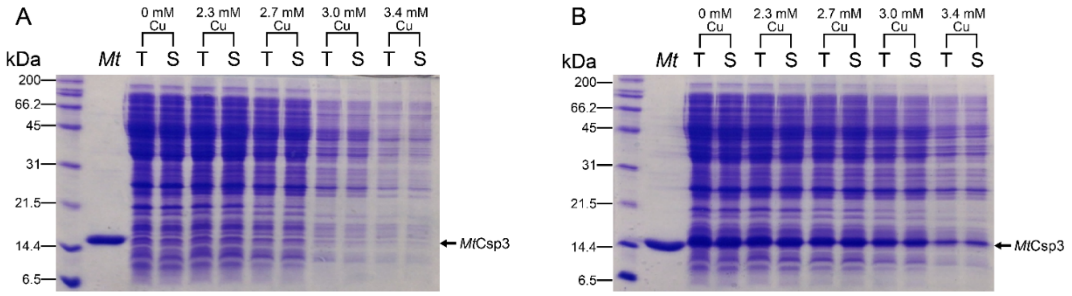

Figure S10. The influence of Cu on the expression levels of *Mt*Csp3 in WT *E. coli*. Analysis by SDS-PAGE of total (T) and soluble (S) proteins in WT *E.coli* plus pBAD33 (A) and pBAD33_*Mtcsp3* (B) after growth for 12 h at different added $Cu(NO_3)_2$ concentrations, compared with a purified sample (15.0 μM) of *Mt*Csp3 (*Mt*).

Table S4. Quantification of *Mt*Csp3 expression levels in WT *E. coli*.

$Cu(NO_3)_2$ (mM)	Proteins	*Mt*Csp3 (μM) [1]
0	Total	17.2 ± 4.62
	Soluble	17.1 ± 3.71
2.3	Total	17.1 ± 3.87
	Soluble	16.9 ± 3.62
2.7	Total	15.6 ± 2.51
	Soluble	17.3 ± 3.79
3.0	Total	13.9 ± 4.74
	Soluble	14.0 ± 4.21
3.4	Total	6.31 ± 1.14
	Soluble	5.73 ± 0.41

[1] The concentrations of *Mt*Csp3 were calculated using the software ImageJ and the average values and standard deviations from three independent growth experiments are shown.

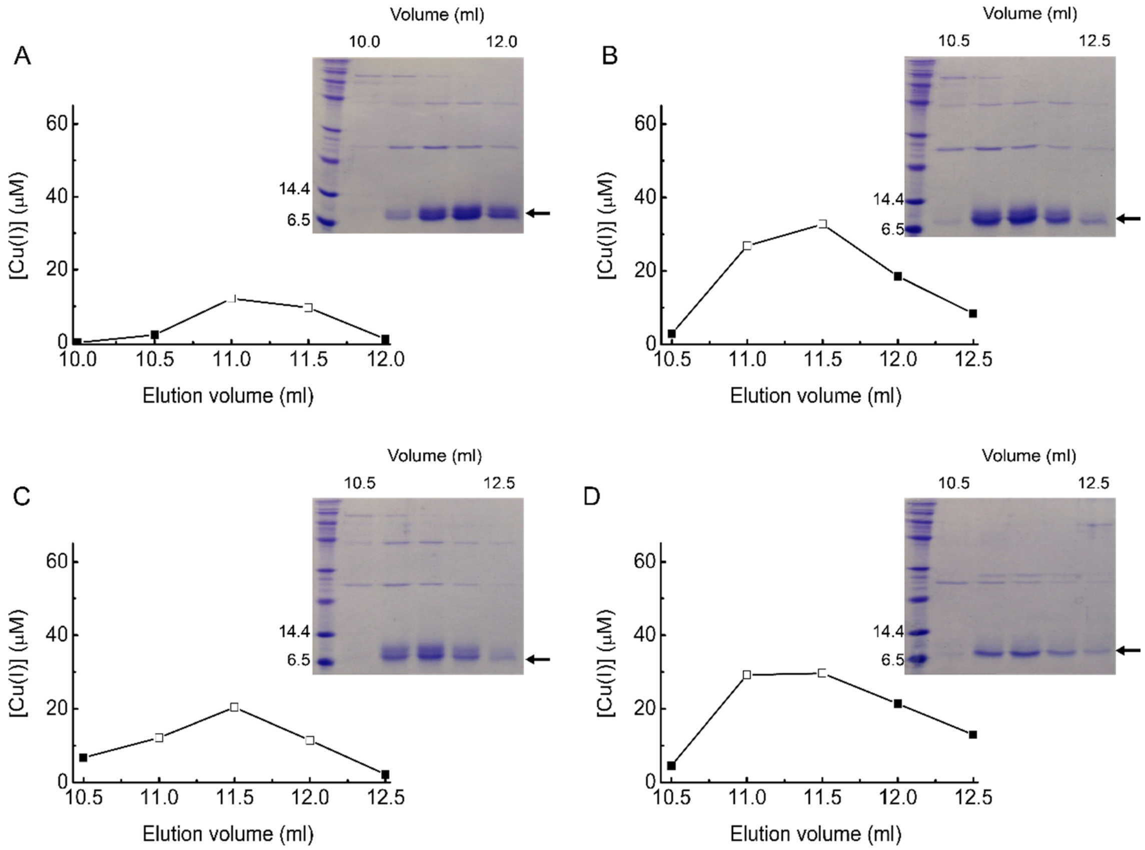

Figure S11. Gel-filtration chromatography of *Bs*Csp3-containing anion-exchange fractions. Plots of Cu(I) concentration against elution volume when the anion-exchange fractions that eluted at 38 mL from cell-free extracts of Δ*copA E. coli* overexpressing *Bs*Csp3 grown in 1.0 (A) and 1.5 (B) mM $Cu(NO_3)_2$ were analyzed by gel-filtration chromatography. Also shown are the gel-filtration analyses of the anion-exchange fractions eluting at 38 and 42 mL when overexpressing *Bs*Csp3 in WT *E. coli* grown in 1.5 (C) and 3.4 (D) mM $Cu(NO_3)_2$, respectively. Insets show SDS-PAGE gels confirming the main protein component in these fractions is *Bs*Csp3 (indicated by an arrow). Open squares identify those fractions that were combined and concentrated for analysis.

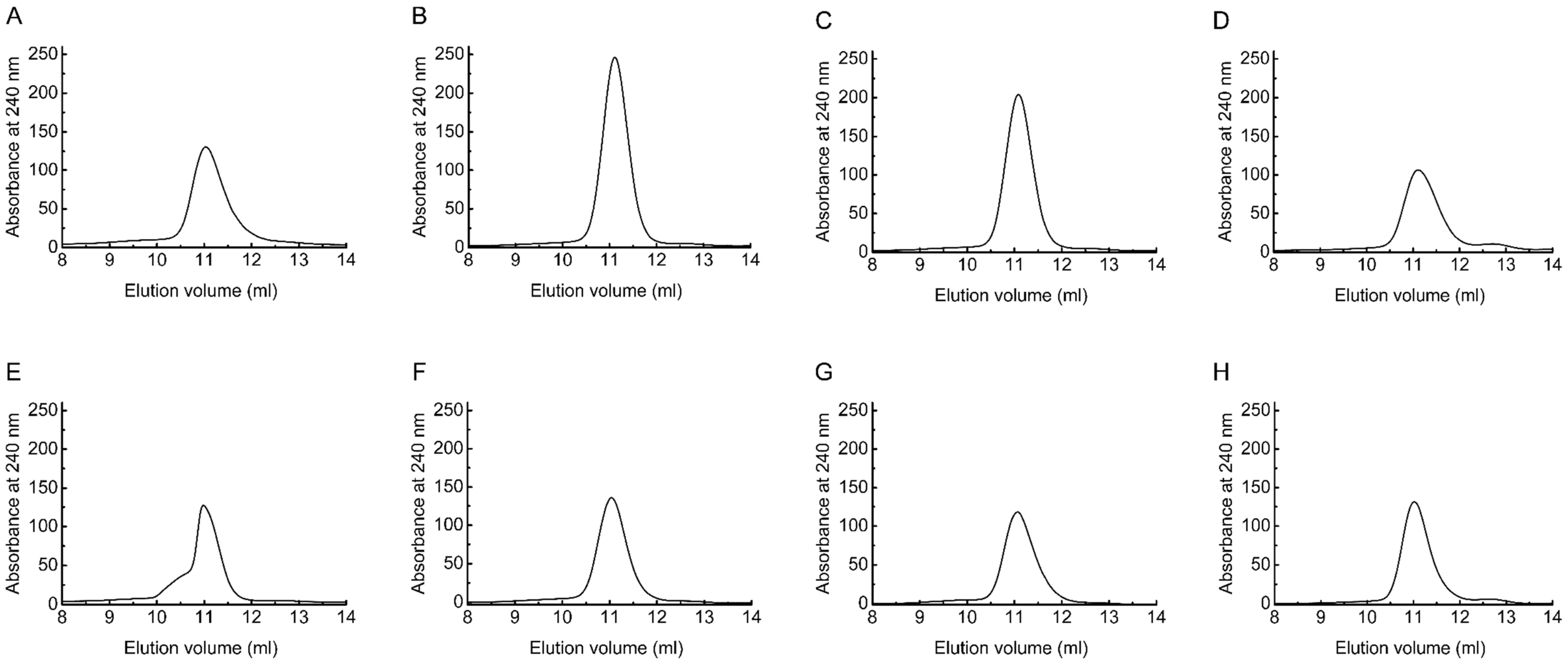

Figure S12. Gel-filtration chromatography of *Bs*Csp3-containing anion-exchange fractions. Plots of absorbance at 240 nm against elution volume when the anion-exchange fractions that eluted at 37 (A) and 38 (E) mL, from Δ*copA E. coli* cells overexpressing *Bs*Csp3 grown in 1.0 mM added $Cu(NO_3)_2$ and those that eluted at 39 (B) and 38 (F) mL when this strain was grown in 1.5 mM added $Cu(NO_3)_2$ were analysed on a Superdex 75 gel-filtration column. Also shown are the corresponding data for the anion-exchange fractions eluting at 39 (C) and 43 (G) mL when overexpressing *Bs*Csp3 in WT *E. coli* grown in 1.5 mM $Cu(NO_3)_2$ and for fractions that eluted at 43 (D) and 42 (H) mL for this strain plus 3.4 mM Cu. The data shown in (A-D) correspond to the gel-filtration chromatograms shown in Figure 7 whilst those in (E-H) are for the chromatograms in Figure S11.

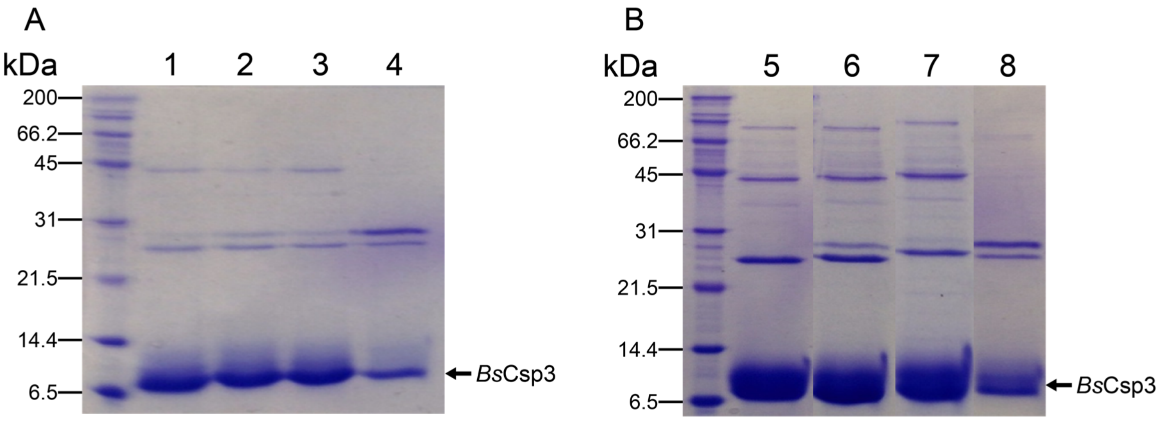

Figure S13. Analysis of the purity of concentrated *Bs*Csp3 samples after gel-filtration chromatography. SDS-PAGE analysis (A) of the concentrated gel-filtration samples from the purification of anion-exchange fractions that eluted at 37 and 39 mL obtained when overexpressing *Bs*Csp3 in Δ*copA E. coli* plus 1.0 and 1.5 mM $Cu(NO_3)_2$, respectively (lanes 1 and 2), and fractions at 39 and 43 mL from WT *E. coli* grown in 1.5 and 3.4 mM $Cu(NO_3)_2$ (lanes 3 and 4). Also shown are the concentrated gel-filtration samples from the purification of anion-exchange fractions that eluted at 38 mL, obtained when overexpressing *Bs*Csp3 in Δ*copA E. coli* plus 1.0 and 1.5 mM $Cu(NO_3)_2$ (lanes 5 and 6), and the fractions at 38 and 42 mL from WT *E. coli* grown in 1.5 and 3.4 mM $Cu(NO_3)_2$, respectively (lanes 7 and 8). The data in (A) correspond to the gel-filtration chromatograms shown in Figure 7 whilst those in (B) are for combined and concentrated samples for the chromatograms in Figure S11.

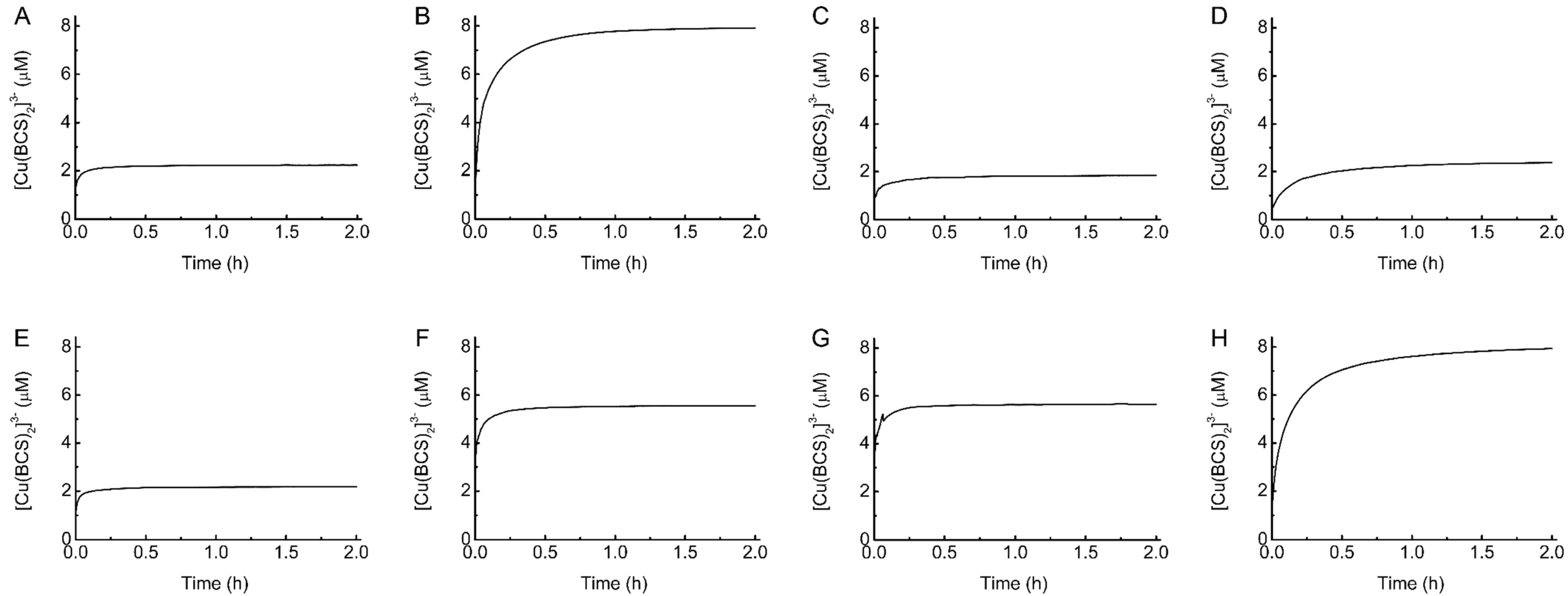

Figure S14. Quantification of Cu(I) in the concentrated *Bs*Csp3-containing gel-filtration samples. Plots of the concentration of the complex $[Cu(BCS)_2]^{3-}$ of the high affinity chromophoric Cu(I) ligand bathcuproine disulfonate (BCS) against time for *Bs*Csp3 from Δ*copA E. coli* grown in 1.0 mM (A) and (E) and 1.5 mM (B) and (F) $Cu(NO_3)_2$, and also *Bs*Csp3 from WT *E. coli* grown in 1.5 mM (C) and (G) and 3.4 mM (D) and (H) $Cu(NO_3)_2$. In (A), (B), (E), (F), (G) and (H) 50 μL of each sample was mixed with 1000 μL of 2.5 mM BCS in 20 mM Hepes pH 7.5 plus 200 mM NaCl and 6.5-6.7 M guanidine hydrochloride. In (C) and (D) 12 μL of sample was used as some precipitation occurred when 50 μL was added.

Table S5. The number of Cu(I) equivalents bound by *Bs*Csp3 from the two *E. coli* strains grown in different amounts of Cu. [1]

E. coli strain and added $Cu(NO_3)_2$ concentration	[Cu(I)] (μM)	[*Bs*Csp3] (μM)	[Cu(I)]/[*Bs*Csp3]
Δ*copA* in 1.0 mM $Cu(NO_3)_2$	47.3	101	0.5
Δ*copA* in 1.5 mM $Cu(NO_3)_2$	117	74.3	1.6
WT in 1.5 mM $Cu(NO_3)_2$	117	96.5	1.2
WT in 3.4 mM $Cu(NO_3)_2$	153	22.2	6.9 [2]

[1] The values shown are the Cu(I) and protein concentrations for *Bs*Csp3 purified by gel-filtration chromatography (Figure S11). [2] The protein concentration is possibly overestimated due the lower purity of this sample (see Figure S13B) and the Cu(I) occupancy of *Bs*Csp3 could therefore be higher than the value quoted.

Table S6. Comparison of the growth of *E. coli* strains overexpressing *Bs*Csp3 in 50 and 500 mL cultures.

<table>
<tr><th>50 mL culture [1]</th><th>OD at 600 nm after 12 h</th><th>[Cu] (μM)/OD</th><th>500 mL culture [2]</th><th>OD at 600 nm after 12 h</th><th>[Cu] (μM)/OD</th></tr>
<tr><td>ΔcopA plus pBAD33_Bscsp3 in 1.0 mM $Cu(NO_3)_2$</td><td>4.31 ± 0.26</td><td>0.87 ± 0.10</td><td>ΔcopA plus pBAD33_Bscsp3 in 1.0 mM $Cu(NO_3)_2$</td><td>4.56</td><td>0.63</td></tr>
<tr><td>ΔcopA plus pBAD33_Bscsp3 in 1.5 mM $Cu(NO_3)_2$</td><td>2.98 ± 0.33</td><td>1.88 ± 0.17</td><td>ΔcopA plus pBAD33_Bscsp3 in 1.5 mM $Cu(NO_3)_2$</td><td>3.44</td><td>1.20</td></tr>
<tr><td>WT plus pBAD33_Bscsp3 in 1.1 mM $Cu(NO_3)_2$</td><td>4.43 ± 0.19</td><td>0.76 ± 0.17</td><td rowspan="2">WT plus pBAD33_Bscsp3 in 1.5 mM $Cu(NO_3)_2$</td><td rowspan="2">5.00</td><td rowspan="2">0.51</td></tr>
<tr><td>WT plus pBAD33_Bscsp3 in 2.3 mM $Cu(NO_3)_2$</td><td>4.24 ± 0.17</td><td>1.02 ± 0.04</td></tr>
<tr><td>WT plus pBAD33_Bscsp3 in 3.4 mM $Cu(NO_3)_2$</td><td>2.29 ± 0.39</td><td>2.31 ± 0.16</td><td>WT plus pBAD33_Bscsp3 in 3.4 mM $Cu(NO_3)_2$</td><td>0.91</td><td>2.77</td></tr>
</table>

[1] Average of three growth experiments. [2] Grown once.

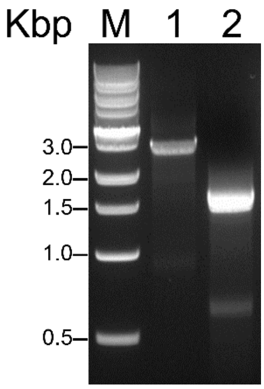

Figure S15. Verification of WT and Δ*copA* BW25113 *E. coli* by PCR. Lane M is a molecular weight marker, whilst the amplified bands for *copA* (2754 bp) in WT *E. coli* and the kanamycin resistance gene in Δ*copA* strain (1571 bp) are present in lanes 1 and 2 respectively.

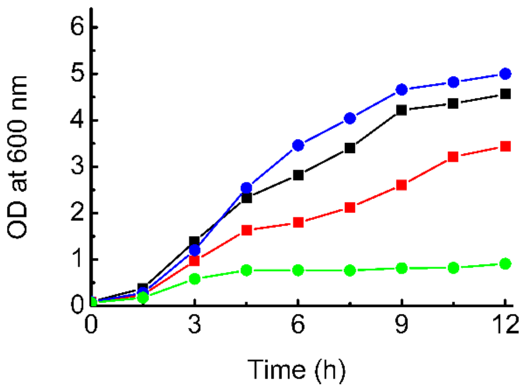

Figure S16. Large scale growth of cells from which *Bs*Csp3 was purified. Cultures (500 mL) of Δ*copA E. coli* plus pBAD33_*Bscsp3* in LB media in the presence of 1.0 (black squares) and 1.5 (red squares) mM $Cu(NO_3)_2$, and WT plus pBAD33_*Bscsp3* in the presence of 1.5 mM (blue circles) and 3.4 (green circles) mM $Cu(NO_3)_2$. The OD values at 12 h and the [Cu] (μM)/OD are shown in Table S6.
